# *In Vitro* Susceptibility of Canine Influenza A (H3N8) Virus to Nitazoxanide and Tizoxanide

**DOI:** 10.4061/2010/891010

**Published:** 2010-08-12

**Authors:** Laura V. Ashton, Robert L. Callan, Sangeeta Rao, Gabriele A. Landolt

**Affiliations:** Department of Clinical Sciences, College of Veterinary Medicine and Biomedical Sciences, Colorado State University, 300 West Drake Road, Fort Collins, CO 80523, USA

## Abstract

Infection of dogs with canine influenza virus (CIV) is considered widespread throughout the United States following the first isolation of CIV in 2004. While vaccination against influenza A infection is a common and important practice for disease control, antiviral therapy can serve as a valuable adjunct in controlling the impact of the disease. In this study, we examined the antiviral activity of nitazoxanide (NTZ) and tizoxanide (TIZ) against three CIV isolates *in vitro*. NTZ and TIZ inhibited virus replication of all CIVs with 50% and 90% inhibitory concentrations ranging from 0.17 to 0.21 *μ*M and from 0.60 to 0.76 *μ*M, respectively. These results suggest that NTZ and TIZ are effective against CIV and may be useful for treatment of canine influenza in dogs but further investigation of the *in vivo* efficacy against CIV as well as the drug's potential for toxicity in dogs is needed.

## 1. Introduction

In 2004, canine influenza virus (CIV) was first isolated from the lung tissue of a racing greyhound dog that had died of pneumonia [1]. Since its first isolation, the virus has spread into the nongreyhound dog population and has caused outbreaks of respiratory disease in dogs throughout the United States [1–3]. As the virus is transmitted in aerosols created by coughing and sneezing, close contact and closed environments favor virus spread. Thus, the highest incidence of infection is found in dogs that are housed in groups, such as at humane shelters [2, 3]. While most dogs infected with CIV have generally mild illness, a small percentage of dogs develop a more severe form of disease characterized by persistent fever, lethargy, dyspnea, and clinical signs of bronchopneumonia [3]. Vaccination is an important practice for the control of influenza infection in many species. Despite the fact that a conditionally licensed, inactivated CIV vaccine has recently become available for use in the United States (Canine Influenza Vaccine, Intervet/Schering-Plough Animal Health, Summit, NJ), it is currently unknown how effective vaccination will be in preventing disease in dogs. Moreover, as effective immunoprotection through active immunization typically requires the administration of several doses of the target antigen (priming and booster doses), vaccination may not represent a realistic approach to confer protection to the transient canine populations housed at humane shelters.

In contrast, antiviral therapy could represent a viable option for the immediate protection of dogs during canine influenza outbreaks, as antiviral drugs do not rely on the induction of a protective immune response [4]. Antiviral agents also represent a potential influenza virus-specific treatment option. A number of animal shelters have employed oseltamivir phosphate (TamiFlu, Roche, Basel, Switzerland) for treatment of dogs during CIV outbreaks. Despite this, the use of oseltamivir phosphate in dogs is not recommended as the dose, duration of treatment, safety, as well as the efficacy of this drug against CIV isolates are unknown [3]. More importantly, oseltamivir should be reserved as a vital defense for the protection of human health during an influenza pandemic. Several studies have demonstrated that the use of antiviral drugs can result in the development of antiviral resistance among influenza A viruses. Indiscriminate use of oseltamivir could potentially reduce the effectiveness of treatment during an influenza pandemic [5–8].

Nitazoxanide (NTZ), a thiazolide compound, has been shown to have antimicrobial activity against a variety of parasites, anaerobic bacteria, and viruses [9–13]. The drug is labeled for treatment of infectious enteritis caused by *Giardia lamblia* and *Cryptosporidium parvum *in humans and for the treatment of equine protozoal myeloencephalitis (EPM) in horses. NTZ has moderate oral bioavailability and, once absorbed, the compound is hydrolyzed in plasma to form the active metabolite tizoxanide (TIZ). NTZ is generally well tolerated and the most commonly reported side effects include mild abdominal pain, nausea, and diarrhea. The compound has been found effective for the treatment of rotavirus and norovirus gastroenteritis in human clinical trials [14, 15]. While the mechanism for its broad-spectrum antiviral activity is not fully understood, it is most likely mediated by a cell-specific rather than a virus-specific effect. This notion is supported by findings of a recent study that demonstrated that NTZ inhibited replication of several human- and avian-lineage influenza A viruses *in vitro* possibly by blocking trafficking of the viral hemagglutinin (HA) protein between the endoplasmic reticulum and the Golgi complex [16]. Taken together, these data suggest that NTZ may be a valuable treatment for dogs infected with CIV, provided that the drug also has inhibitory activity against canine influenza viruses. In this report, we present results of *in vitro* studies characterizing the activities of NTZ and TIZ against three recent canine influenza virus isolates.

## 2. Materials and Methods

### 2.1. Influenza Viruses

Field isolates of A/Canine/Colorado-1/224986/06 (Ca/CO-1), A/Canine/Colorado-3/3/06 (Ca/CO-3), and A/Canine/Colorado-4/2025974/07 (Ca/CO-4) (all H3N8) were cultivated in Madin-Darby canine kidney (MDCK) cells as previously described in [17]. To confirm sequence identity, the full-length protein coding regions of the HA and neuraminidase (NA) genes of Ca/CO-1, Ca/CO-3, and Ca/CO-4 were amplified by RT-PCR and sequenced as previously described in [18].

### 2.2. Determination of Cell Toxicity

Nitazoxanide (NTZ) was provided by IDEXX Laboratories (Westbrook, ME, USA) and tizoxanide (TIZ) was provided by Romark Laboratories (Tampa, FL, USA). Stock solutions were prepared by dissolving 50 mg NTZ or TIZ in 1 mL of dimethyl sulfoxide (DMSO), divided into aliquots, and stored at −80°C. Working solutions of NTZ and TIZ were prepared fresh by diluting the stock solution to the appropriate *μ*M concentration in MEM supplemented with 100 IU penicillin/streptomycin, 100 mM L-glutamine, 0.5% bovine serum albumin (GIBCO, Carlsbad, CA, USA) and 1 *μ*g/mL tolylsulfoyl phenylalanyl choromthyl ketone-(TPCK-) treated trypsin (Worthington Biochemical Corporation, Lakewood, NJ, USA). Working solutions of NTZ or TIZ were then added to a confluent monolayer of MDCK cells in 96-well tissue culture plates (BD Biosciences, San Jose, CA, USA). Negative (no NTZ or TIZ) and DMSO (1%) control samples were included in each experiment. Plates were then incubated at 37°C with 5% CO_2_. Cell cytotoxicity of NTZ and TIZ was determined after 96 hours using the alamar blue reduction assay by methods previously described [19]. Cell viability was expressed as the CC_50_, which is the Cytotoxic Concentration at which a 50% decrease in alamar blue reduction was noted when compared to the negative control.

### 2.3. Determination of NTZ and TIZ Inhibitory Concentrations

Confluent monolayers of MDCK cells cultured in 96-well tissue culture plates were infected at a multiplicity of infection (MOI) of 0.001 with Ca/CO-1, Ca/CO-3, or Ca/CO-4. Infected plates were incubated with agitation for 2 hours at 37°C with 5% CO_2_. After incubation, the virus inoculum was removed and 200 *μ*L of working solutions of NTZ or TIZ, at concentrations ranging from 0.001 *μ*M to 4.0 *μ*M, were added to each well. Positive (no NTZ or TIZ) and negative (no virus) control samples as well as a 1% DMSO control were included in each experiment. Plates were then incubated at 37°C with 5% CO_2_ for 72 hours. The virus titer in cell culture supernatants of each well, expressed as tissue culture infective dose (TCID)_50_ per mL, was calculated using the method of Reed and Muench as previously described [17].

### 2.4. Statistical Analysis

Each experiment was done on 3 different occasions with 8 samples of each NTZ or TIZ concentration per replicate (*n* = 24). The amount of infectious CIV in the treated culture supernatant was expressed as a percentage of the control value and was calculated by dividing the titer of each treated sample by the average of the untreated positive control. To normalize the data, the percentage control values were log transformed. The data was analyzed by polynomial regression analysis. Based on the fit and overall variance (*R* ^2^), the best model was selected to determine the antiviral effective concentrations that result in the 50% (EC_50_) and 90% (EC_90_) reduction of virus titers in the culture supernatants. Selectivity index (SI) was calculated by dividing the CC_50_ by the EC_50_. To evaluate the association between the independent variables (dose, drug, and strain) and the outcome variable (viral titer relative to the control), the data was analyzed a multivariable linear regression analysis with a mixed model approach PROC MIXED (SAS v9.1, SAS Institute Incorporated, Cary, NC) by specifying the fixed and random effects. A critical alpha for all statistical analyses was set *a priori* at 0.05.

## 3. Results

**Figure 1:**
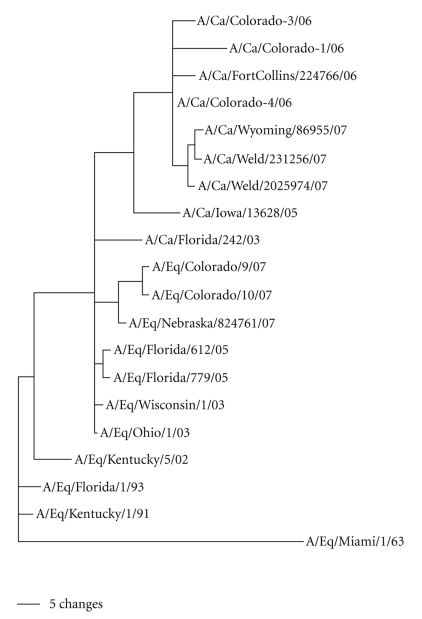
Phylogenetic relationships at the nucleotide level among the hemagglutinin 3 (H3) genes of contemporary canine (including Ca/CO-1, Ca/CO-3, and Ca/CO-4 used in this study) and equine influenza viruses. Scale bar represents a difference at the nucleotide level of 5%.

Sequencing and phylogenetic analyses of the HA and NA genes of the three canine influenza isolates used in this study confirmed that Ca/CO-1, Ca/CO-3, and Ca/CO-4 clustered with the contemporary canine H3N8 influenza viruses, placing them into the previously described distinct canine sublineages of the equine H3 “Florida lineage” (Figure 1) [2]. NTZ and its active metabolite TIZ exhibited dose-dependent inhibition of extracellular virus production by MDCK cells. Complete inhibition of *in vitro* replication of Ca/CO-1, Ca/CO-3, and Ca/CO-4 in MDCK cells in the presence of NTZ or TIZ was observed at a concentration of 5 *μ*M. At concentrations of NTZ or TIZ between 1 and 2 *μ*M, replication of all three CIV isolates was partially inhibited resulting in the reduction of virus titers by 3 log_10_ compared to the control wells.

**Figure 2:**
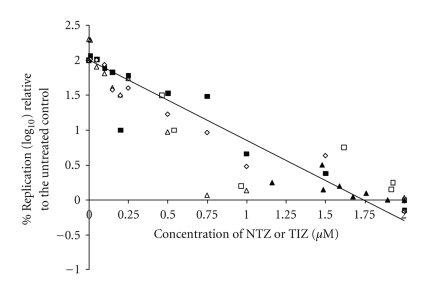
Effect of NTZ (closed) or TIZ (open) against CIV (Ca/CO-1 (▲, Δ) Ca/CO-3 (■, □) and Ca/CO-4 (♦, *◊*)) relative to the replication of the untreated controls in MDCK cells.

To determine EC_50_ and EC_90_, assays were performed using NTZ or TIZ concentrations ranging from 0.001 to 2 *μ*M. Dose response curves for Ca/CO-1, Ca/CO-3, and Ca/CO-4 viral titers are shown in Figure 2. The best model selected with good fit and 82% overall variance (*R* ^2^) was the Quadratic polynomial model. The multivariable linear regression analysis, the selected model for the viral titers with CIV strain Ca/CO-4 was significantly higher (*P* < .05) than Ca/CO-1. The viral titers with CIV strain Ca/CO-4 were lower than Ca/CO-1 and Ca/CO-3 with *P* = .07. However, this did not result in significant differences between the EC_50_ and EC_90_ for the three viral isolates (Table 1). The model also indicated that the viral titers with addition of TIZ were significantly higher (*P* < .05) compared to those with addition of NTZ. While the EC_50_ values for NTZ and TIZ for the three CIV isolates (0.17–0.21 *μ*M) were similar for each strain (Table 1), the EC_90_ values were consistently 3.5 times higher than the EC_50_ (0.60–0.76 *μ*M) regardless of strain or drug form (NTZ or TIZ). In contrast, the selectivity index of TIZ against all three CIV strains was double the value obtained for the parent compound NTZ (Table 1). This was directly related to the CC_50_ values for NTZ and TIZ on MDCK cells, which were 64.9 *μ*M and 130 *μ*M, respectively.

## 4. Discussion

The recent emergence of canine influenza virus represents a serious threat to canine health and welfare. As dogs possess no natural immunity against this virus, all dogs, regardless of age, breed, or sex are potentially susceptible to infection. While the total number of CIV infections that have occurred since the appearance of the virus in 2004 is unknown, a recent one-year study performed by our laboratory found CIV PCR-positive dogs in 11 of 16 (69%) Colorado humane shelters that experienced outbreaks of canine respiratory disease [20]. These results support the importance of CIV as a cause of morbidity in shelter dogs in the United States. Despite the fact that an inactivated vaccine has recently become available, antiviral therapy may be beneficial in fluctuating populations such as in shelters because initial vaccination series for the canine vaccine should include two doses. In this study, we report that the thiazolide NTZ and its active metabolite TIZ possess potent antiviral activities against three genetically distinct canine influenza viruses. Inhibition of virus replication was dose dependent and the concentrations of NTZ and TIZ needed to inhibit CIV replication were comparable to the concentrations found to be effective against Hepatitis B Virus and Hepatitis C Virus (HCV) (0.12–0.21 *μ*M) [21] but lower than the concentrations needed to inhibit several human influenza viruses and an avian isolate (1.63–3.25 *μ*M) [16]. Several studies indicate that concentrations necessary to inhibit CIV replication are readily achievable in plasma following oral administration of NTZ to humans [22, 23]. In humans, the drug is typically well tolerated and only mild, and generally transient, adverse effects have been reported [23, 24]. In contrast, since the drug is also active against microaerophilic and anaerobic bacteria that are a part of the normal gastrointestinal flora, substantial side effects, such as enterocolitis, have been described in horses following administration of NTZ [25]. Since cytotoxic levels observed *in vitro* do not necessarily indicate the actual toxicity of NTZ in the animal, further research is needed to determine the pharmacokinetics and safety of NTZ in dogs.

Currently, two classes of antiviral agents are available for prophylaxis and treatment of influenza A virus infections; the M2 ion channel blockers and the neuraminidase inhibitors. The M2 ion channel blockers amantadine and rimantadine inhibit virus replication through allosteric inhibition of the M2 protein, thus blocking the flow of H^+^ ions from the acidified endosomal compartment into the interior of the virion. The neuraminidase inhibitors, oseltamivir phosphate, and zanamivir block the active site of the viral neuraminidase protein preventing the release of the influenza virion from infected cells. Of these antivirals, oseltamivir phosphate is currently considered the drug of choice for treatment of influenza in humans. However, as resistance to oseltamivir and the M2 ion channel blockers can be conferred by a single point mutation [26–28], antiviral resistance among influenza A isolates is rapidly increasing [6, 29–31]. As the viral RNA polymerase lacks proofreading function, influenza A viruses demonstrate high mutations rates. Given the plasticity of the influenza virus genome, selective pressures will inevitably lead to mutational changes.

Unlike direct-acting antiviral drugs, NTZ does not inhibit viral RNA transcription or block viral protein synthesis, instead, the drug interferes with cellular posttranscriptional protein processing and may interfere with the assembly of viral glycoproteins, preventing the formation of mature viral particles [9, 21]. For example, NTZ has been shown to inhibit the function of protein disulfide isomerase [32], an important enzyme for three-dimensional protein formation in the endoplasmic reticulum (ER). More specifically, Rossignol and colleagues demonstrated that NTZ exerts its anti-influenza effects by interfering with the maturation and the intracellular trafficking of the HA protein [16]. Given the drug's activity against a wide range of viral pathogens, it has been speculated that NTZ's antiviral effect is mediated by a cell-specific rather than a virus-specific mechanism [16]. This hypothesis is supported by the finding that transfection of HCV viral RNA isolated from NTZ or TIZ-resistant replicon-containing cell lines did not transfer NTZ or TIZ resistance, suggesting an absence of acquired resistance in the HCV genome [33]. Taken together, this suggests that the potential for the development of antiviral resistance to NTZ by mutational adaptation of the virus genome may be minimal.

## 5. Conclusions

Our results represent the first report of the *in vitro* activity of NTZ and its active metabolite TIZ against three genetically distinct, contemporary canine influenza viruses. The use of a broad-spectrum antiviral such as NTZ in conjunction with supportive care may reduce the morbidity and risk of secondary complications associated with canine influenza. The use of NTZ as a prophylactic and therapeutic antiviral during CIV outbreaks could possibly shorten the duration of nasal virus shedding, thus limiting the spread of the virus. However, further investigation of the *in vivo* efficacy against CIV as well as the drug's potential for toxicity in dogs is needed.

## Figures and Tables

**Table 1:** *In vitro* activity of nitazoxanide (NTZ) and its active metabolite tizoxanide (TIZ) on clinical isolates of canine influenza virus (CIV).

Virus	Drug	EC_50_	EC_90_	SI
Ca/CO-1	NTZ	0.20 ± 0.01	0.70 ± 0.03	331
	TIZ	0.20 ± 0.01	0.72 ± 0.03	644

Ca/CO-3	NTZ	0.17 ± 0.01	0.60 ± 0.03	391
	TIZ	0.17 ± 0.01	0.62 ± 0.03	751

Ca/Co-4	NTZ	0.20 ± 0.01	0.71 ± 0.03	329
	TIZ	0.19 ± 0.01	0.70 ± 0.03	374

CIV	NTZ	0.21 ± 0.02	0.76 ± 0.03	309
	TIZ	0.21 ± 0.02	0.75 ± 0.03	619

EC_50  or 90_: Concentration (*μ*M) of NTZ or TIZ at which a 2-fold (50%) or 10-fold (90%) reduction of viral titer is relative to untreated controls. EC_50_ and EC_90_ are expressed as means ± standard deviation.

SI: Selectivity Index (CC_50_/EC_50_).

Viral titers of three CIV isolates were analyzed using polynomial regression analysis. 95% confidence intervals for each value are displayed in parenthesis.
